# Threads of Elasticity: A Single Variant Journey Through Pseudoxanthoma Elasticum’s Clinical Maze

**DOI:** 10.7759/cureus.89237

**Published:** 2025-08-02

**Authors:** Divyashri R Nagarajan, Daya Mani Jacob, Sathya Kakade

**Affiliations:** 1 Ophthalmology, Burjeel Medical City, Abu Dhabi, ARE; 2 Internal Medicine, Burjeel Medical City, Abu Dhabi, ARE

**Keywords:** abcc6 gene mutation, angioid streaks, choroidal neovascularization, monoallelic variant, pseudoxanthoma elasticum

## Abstract

We present a case of a 23-year-old female with characteristic skin papules and angioid streaks characteristic of pseudoxanthoma elasticum (PXE), an autosomal recessive disorder of elastic fiber mineralization. Genomic sequencing revealed a heterozygous variant in the ABCC6 gene. Despite the absence of biallelic mutations, the clinical phenotype aligns with PXE. Our focus in this report is to highlight the importance of multidisciplinary assessment, genotype-phenotype correlation, and the diagnostic role of ophthalmic findings in patients with atypical or partial PXE presentations.

## Introduction

Pseudoxanthoma elasticum (PXE), also called Gronblad-Strandberg syndrome, is a rare, inherited connective tissue disorder caused by pathogenic variants in the ABCC6 gene, resulting in calcification and fragmentation of elastic fibers in the skin, retina, and vasculature [1].

PXE has an estimated prevalence of one in 50,000 in the general population. Based on this prevalence, it is estimated to affect around 150,000 individuals worldwide. The condition is more frequently seen in females than males, with a ratio of 2:1. Clinical signs typically do not appear at birth but usually emerge during the second or third decade of life [2].

Clinically, PXE manifests with yellowish papular skin lesions, angioid streaks of the retina, and progressive visual impairment, often accompanied by cardiovascular complications and gastrointestinal bleeding [1,2]. The papular skin lesions are small yellow papules on the nape and sides of the neck and in flexural areas. The papules coalesce, and the skin becomes loose and wrinkled. The mid-dermal elastic fibers are short, fragmented, clumped, and calcified. Dystrophic calcification of Bruch’s membrane, revealed by angioid streaks, may trigger choroidal neovascularization and, ultimately, loss of central vision and blindness in late-stage disease. Lesions in small and medium-sized artery walls may result in intermittent claudication and peripheral artery disease. Cardiac complications such as myocardial infarction and angina pectoris are thought to be relatively rare but merit thorough investigation [1,3].

There is currently no cure for PXE. Symptomatic treatment focuses on managing specific complications and includes therapies such as vascular endothelial growth factor inhibitors for eye involvement, lifestyle modifications, lipid-lowering medications, and dietary changes to reduce vascular risk, as well as vascular surgery in cases of severe cardiovascular disease. Emerging treatment approaches being explored for the future include gene therapy, gene editing, and pharmacologic chaperone therapy [3].

The classic inheritance pattern is autosomal recessive, requiring biallelic mutations in the ABCC6 gene. However, emerging literature recognizes the phenotypic overlap in heterozygous carriers, raising questions about incomplete penetrance and modifier genes [3].

This case report aims to highlight the diagnostic importance of ophthalmologic findings, particularly angioid streaks, in identifying PXE, even in patients with only one identified ABCC6 variant. By sharing this case, we hope to raise clinical awareness of PXE and encourage early recognition and timely genetic testing for appropriate diagnosis and management.

## Case presentation

A 23-year-old female presented with longstanding dermatologic complaints, including itchy, yellowish-pink papules localized to the neck, axillae, and groin. These lesions had been present since childhood and were persistent and moderately severe, sometimes painful. Her medical history was notable for epilepsy, which began in 2019 and has since been managed effectively with antiepileptic medications. Additionally, she had a recorded total cholesterol level of 240 mg/dL on a lipid panel, indicating hypercholesterolemia.

The patient’s family history revealed that her parents were first cousins. She had five siblings, one of whom had short stature. There was no known family history of similar skin, eye, or systemic findings. Given the presence of multiple findings and a consanguineous background, she was referred for genetic counseling. Whole genome sequencing revealed a heterozygous variant of uncertain significance (VUS) in the ABCC6 gene. Although PXE is typically inherited in an autosomal recessive manner, requiring biallelic mutations for definitive molecular diagnosis, the patient’s clinical presentation raised strong suspicion for the disorder. Importantly, the strong phenotypic correlation in this patient may support future reclassification of this VUS as likely pathogenic. Segregation analysis for other family members was recommended to further assess the pathogenicity of the variant.

**Figure 1:**
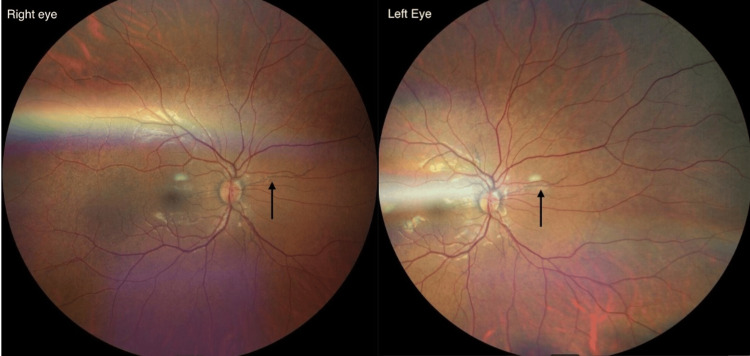
Fundoscopy showing bilateral angioid streaks.

Due to complaints of blurred vision, the patient was referred for a full ophthalmologic evaluation. The best corrected visual acuity was 6/6. Fundoscopic examination revealed bilateral angioid streaks (Figure 1) and linear breaks in Bruch’s membrane radiating from the optic disc, consistent with PXE. The macula was normal, with a healthy foveal reflex and no drusen or edema.

The combination of dermatologic findings, bilateral angioid streaks, and the presence of a heterozygous ABCC6 variant raised a strong suspicion for PXE. Although the genetic diagnosis could not be molecularly confirmed due to the monoallelic nature of the variant, the clinical presentation supports the diagnosis. The patient was counseled on the systemic implications of PXE and was referred to a multidisciplinary team, including dermatology, cardiology, gastroenterology, neurology, and ophthalmology, for ongoing surveillance and management.

## Discussion

**Figure 2:**
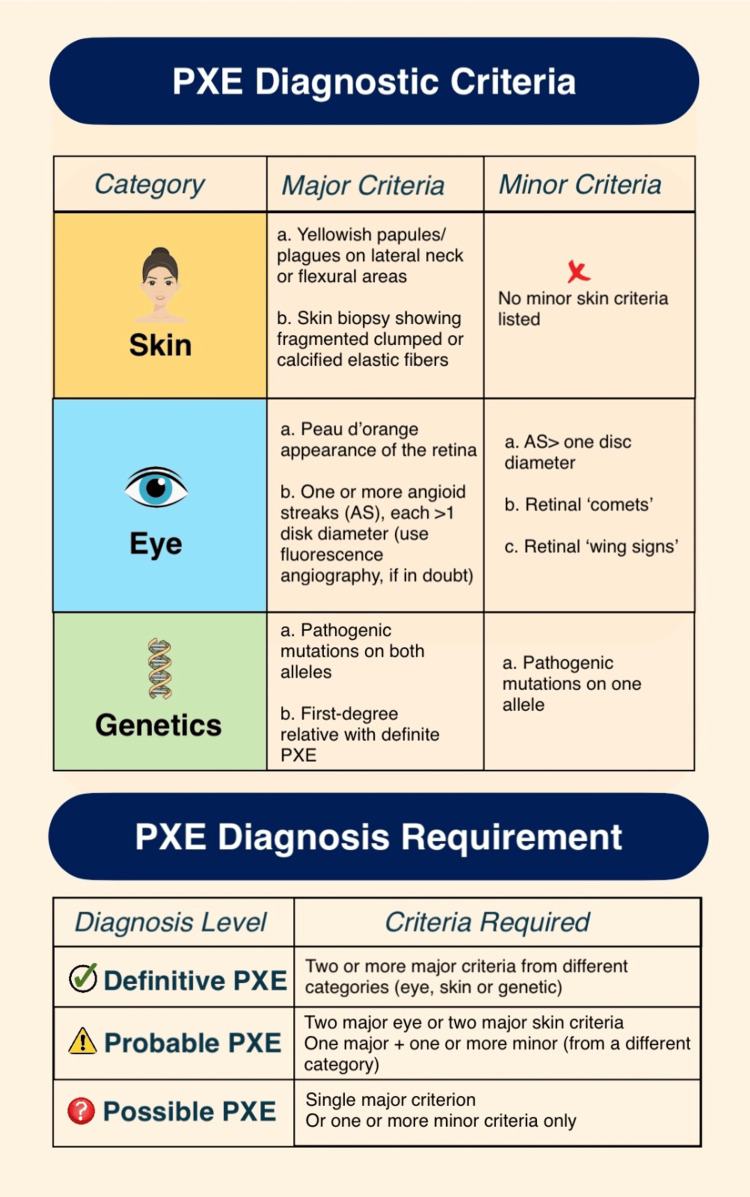
Diagnostic criteria for pseudoxanthoma elasticum (PXE). Figure credits: Divyashri R. Nagarajan. Source: Germain [3].

The patient presented with yellowish-pink papules on the neck, angioid streaks in the eye, and a pathologic mutation of ABCC6 in one allele, which fulfills the two major and one minor diagnostic criteria for PXE, according to Figure 2.

This patient meets the clinical diagnostic criteria for PXE, based primarily on characteristic dermatologic and ophthalmologic findings, most notably, bilateral angioid streaks, a hallmark of PXE-associated retinal pathology [1]. These streaks result from breaks in Bruch’s membrane caused by calcified elastic fibers and can predispose to choroidal neovascularization and progressive vision loss [2].

The management of PXE is inherently multidisciplinary and largely supportive, targeting its cutaneous, ocular, and vascular manifestations. While the yellow, cobblestone-like skin lesions typical of PXE are often benign and primarily pose aesthetic concerns, they can be diagnostically useful and frequently prompt screening for ABCC6 gene mutations. Surgical intervention for skin manifestations should be approached with caution, as these symptoms are not life-threatening [3].

Ocular complications, particularly choroidal neovascularization, are a significant source of morbidity. Intravitreal anti-VEGF therapies, such as bevacizumab, have become the mainstay of treatment, often superseding older modalities like photodynamic therapy. Patients are generally advised to avoid contact sports due to the risk of retinal hemorrhage and detachment. Consequently, regular surveillance by a retina specialist is essential to monitor and manage ocular complications [4].

Vascular involvement, including the development of arterial aneurysms, a major contributor to PXE-related morbidity [5], requires proactive risk reduction strategies. These include smoking cessation, regular physical activity, and weight management [3]. While cholesterol-lowering agents such as atorvastatin may be beneficial, antiplatelet agents like aspirin are typically avoided due to heightened risks of retinal or gastrointestinal bleeding. In more advanced cases, surgical or endovascular intervention may be warranted for arterial stenosis, though thorough preoperative assessment is critical given the structural fragility of affected vessels [3].

Several experimental therapies are under investigation. Dietary magnesium supplementation and bisphosphonates (e.g., etidronate) have demonstrated some success in animal models by reducing ectopic mineralization [5].

Additionally, correction of protein misfolding using sodium 4-phenylbutyrate has shown potential to restore ABCC6 function and reduce calcification, though only for specific mutations and in early-stage research. Gene therapy offers another promising avenue, with liver-directed delivery systems aiming to restore ABCC6 expression [5]. Although no curative treatment currently exists for PXE, a combination of targeted symptomatic management and emerging experimental strategies holds promise for altering the disease trajectory in the future.

Genetic counseling played a vital role in this case, outlining the autosomal recessive inheritance pattern, recommending segregation analysis in family members, and discussing reproductive options such as prenatal and preimplantation genetic testing in the presence of a second confirmed mutation [6,7].

This case gives a learning perspective that clinical diagnosis of PXE should not be excluded in the presence of a heterozygous ABCC6 variant, especially when classic phenotypic features such as angioid streaks and characteristic skin lesions are present, and in patients from consanguineous backgrounds with classic phenotypic features. The strong genotype-phenotype correlation raises the possibility of a second, undetected pathogenic variant not identified by standard sequencing methods. This proposes the need for consideration of more advanced techniques, such as long-read sequencing or RNA analysis, in unresolved cases. It also reflects the importance of re-evaluating VUS classifications over time as new clinical and genetic data emerge. Ophthalmologic findings remain a cornerstone of diagnosis, and comprehensive multidisciplinary management, including genetic counseling, even in the absence of biallelic mutations, is essential for guiding family studies, anticipatory care, and long-term planning.

## Conclusions

This case underscores the diagnostic challenge of PXE in the setting of a heterozygous ABCC6 variant, with classic phenotypic features and a consanguineous family background. A multidisciplinary approach involving dermatology, ophthalmology, cardiology, and genetics is essential for optimal diagnosis, surveillance, and counseling. Given the strong phenotype despite a single detected mutation, future studies should explore whether such cases warrant deeper genetic analysis to identify a potential second variant. This report also contributes to growing evidence of partial phenotypic expression in heterozygous ABCC6 carriers and reinforces the importance of long-term follow-up and familial evaluation.
